# (*E*)-2-Amino-4-(3,3-dimethyl-2-oxobutyl­idene)-1-[2-(2-hy­droxy­eth­oxy)eth­yl]-6-methyl-1,4-dihydro­pyridine-3-carbo­nitrile

**DOI:** 10.1107/S160053681203334X

**Published:** 2012-07-28

**Authors:** Hyung Jin Kim, Young Hyun Kim, Enkhzul Otgonbaatar, Chee-Hun Kwak

**Affiliations:** a School of Applied Chemical Engineering, Chonnam National University, Gwangju 500-757, Republic of Korea; b Department of Chemistry, Sunchon National University, Sunchon 540-742, Republic of Korea

## Abstract

In the title compound, C_17_H_25_N_3_O_3_, there are intra­molecular hydrogen bonds between an amine H atom and the ep­oxy O atom, and between a dihydro­pyridine ring H atom and the ketone O atom. In the crystal, mol­ecules are linked into a zigzag chain running parallel to the *c* axis by hydrogen bonds between the hy­droxy group and the ketone O atom. There are also weak C—H⋯O and C—H⋯π inter­actions which link the mol­ecules into sheets lying in the *bc* plane.

## Related literature

For related structures, see: Ha *et al.* (2009 ▶); Kim *et al.* (2011 ▶). For the synthesis, see: VanAllan & Reynolds (1971 ▶).
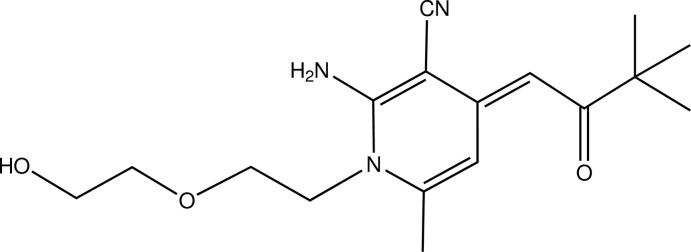


## Experimental

### 

#### Crystal data

C_17_H_25_N_3_O_3_
*M* *_r_* = 319.40
Monoclinic, 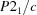

*a* = 9.9007 (5) Å
*b* = 13.2890 (7) Å
*c* = 13.0686 (8) Å
β = 91.314 (2)°
*V* = 1718.99 (16) Å^3^
*Z* = 4
Mo *K*α radiation
μ = 0.09 mm^−1^
*T* = 293 K
0.6 × 0.4 × 0.2 mm

#### Data collection

Rigaku R-AXIS RAPID II-S diffractometer
Absorption correction: multi-scan (*RAPID-AUTO*; Rigaku, 2008 ▶) *T*_min_ = 0.960, *T*_max_ = 0.983
15989 measured reflections
3912 independent reflections
3104 reflections with *I* > 2σ(*I*)
*R*_int_ = 0.058

#### Refinement

*R*[*F* ^2^ > 2σ(*F* ^2^)] = 0.049
*wR*(*F* ^2^) = 0.136
*S* = 1.08
3912 reflections
209 parameters
H-atom parameters constrained
Δρ_max_ = 0.36 e Å^−3^
Δρ_min_ = −0.26 e Å^−3^

### 

Data collection: *RAPID-AUTO* (Rigaku, 2008 ▶); cell refinement: *RAPID-AUTO*; data reduction: *RAPID-AUTO*; program(s) used to solve structure: *SHELXS97* (Sheldrick, 2008 ▶); program(s) used to refine structure: *SHELXL97* (Sheldrick, 2008 ▶); molecular graphics: *ORTEP-3* (Farrugia, 1997 ▶); software used to prepare material for publication: *SHELXL97*.

## Supplementary Material

Crystal structure: contains datablock(s) I, global. DOI: 10.1107/S160053681203334X/go2062sup1.cif

Structure factors: contains datablock(s) I. DOI: 10.1107/S160053681203334X/go2062Isup2.hkl

Supplementary material file. DOI: 10.1107/S160053681203334X/go2062Isup3.cml

Additional supplementary materials:  crystallographic information; 3D view; checkCIF report

## Figures and Tables

**Table 1:** Hydrogen-bond geometry (Å, °) *Cg*1 is the centroid of the dihydro­pyridine ring.

*D*—H⋯*A*	*D*—H	H⋯*A*	*D*⋯*A*	*D*—H⋯*A*
N3—H3*A*⋯O1	0.86	2.27	2.9083 (17)	131
C4—H4⋯O3	0.93	2.21	2.8405 (17)	124
O2—H2⋯O3^i^	0.82	2.03	2.650 (2)	132
C7—H7*A*⋯O2^ii^	0.97	2.47	3.303 (2)	144
C9—H9*A*⋯*Cg*1^ii^	0.97	2.81	3.5275 (16)	131

Symmetry codes: (i) 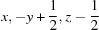; (ii) 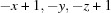.

**Fig. 1.**
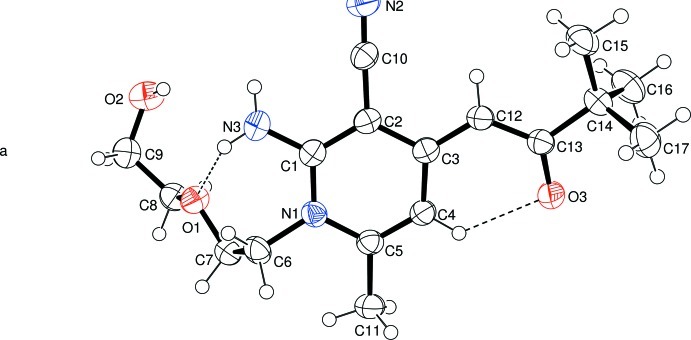
The structure of titled compound with displacement ellipsoids drawn at 50% probability level for non-H atom.

**Fig. 2.**
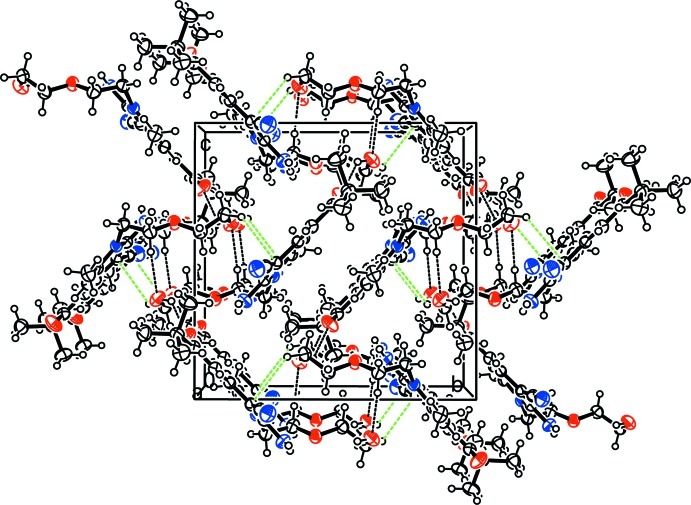
A packing diagram of the titled compound. Dashed line are intermolecular hydrogen bonds (black) and C—H···π interactions (green).

## supplementary crystallographic information

### Comment

2,6-Disubstituted (1,4-pyridine-4-ylidene)malononitrile derivatives are key intermediates in the synthesis of dihydropyridine-based fluorescent dyes (Ha *et al.*, 2009; Kim *et al.*, 2011), and are readily prepared from the reaction of the corresponding 2,6-disubstituted (4*H*-pyran-4-ylidene)maolononitrile with a primary amine. We reacted 2-(2-*tert*-butyl-6-methyl-4*H*-pyran-4-ylidene)malononitrile with 2-(2-aminoethoxy)ethanol in order to obtain the (1,4-pyridine-4-ylidene)malononitrile substituted with *tert*-butyl and methyl groups at 2 and 6-positions, respectively. The title compound was however produced as a major product instead of the corresponding malononitrile derivative (VanAllan & Reynolds, 1971). The molecular structure of C_17_H_25_N_3_O_3_, is shown in (Fig. 1). There are intramolecular hydrogen bonds N3-H3···O1 and C4–H4···O3 (Table 1). An intermolecular hydrogen bond O2-H2···O3(*x*,1/2-*y*,-1/2+ *z*) links the molecules into a zigzag chain which runs along the *c* axis. These chains are linked to form a sheet by a weak C7–H7A···O21-*x*,-y,1-*z* and as C9—H9A···π interaction involving the dihydropyridine ring(1-*x*,-y,1-*z*) 1, (Table 1, Fig. 2).

### Experimental

A mixture of 2-(2-*tert*-butyl-6-methyl-4*H*-pyran-4-ylidene)malononitrile (632 mg, 2.94 mmol) and 2-(2-aminoethoxy)ethanol (465 mg, 4.42 mmol) dissolved in *n*-butanol (7 ml) was heated at 100 °C for 4 h. The mixture was cooled and concentrated under vacuum. The residue was chromatographed on SiO_2_ eluting with a mixture of EtOAc/MeOH (1:1) solution to afford the title compound (300 mg, 32%) as a yellow solid. Crystals suitable for X-ray analysis were obtained by slow evaporation from a CHCl_3_/MeOH solution at room temperature. Mp 173 °C.

### Refinement

H atoms were positioned geometrically and allowed to ride on their respective parent atoms [C—H = 0.93 (CH, *sp*^2^), 0.98 (CH, *sp*^3^), 0.86 (NH_2_) and 0.82 Å (OH), respectively and *U*_iso_(H) = 1.2*U*_eq_(C), 1.2*U*_eq_(N) and 1.5*U*_eq_(O), respectively]. The positions of the methyl, amino and hydroxyl H atoms were checked on a final difference map and were found to be satisfactory.

### Crystal data

**Table:**

C_17_H_25_N_3_O_3_	*F*(000) = 688
*M**_r_* = 319.40	*D*_x_ = 1.234 Mg m^−^^3^
Monoclinic, *P*2_1_/*c*	Mo *K*α radiation, λ = 0.71073 Å
*a* = 9.9007 (5) Å	θ = 3.0–27.5°
*b* = 13.2890 (7) Å	µ = 0.09 mm^−^^1^
*c* = 13.0686 (8) Å	*T* = 293 K
β = 91.314 (2)°	Block, brown
*V* = 1718.99 (16) Å^3^	0.6 × 0.4 × 0.2 mm
*Z* = 4	

### Data collection

**Table:**

Rigaku R-AXIS RAPID II-S diffractometer	3912 independent reflections
Radiation source: fine-focus sealed tube	3104 reflections with *I* > 2σ(*I*)
Graphite monochromator	*R*_int_ = 0.058
ω scans	θ_max_ = 27.5°, θ_min_ = 3.0°
Absorption correction: multi-scan (*RAPID-AUTO*; Rigaku, 2008)	*h* = −12→12
*T*_min_ = 0.960, *T*_max_ = 0.983	*k* = −17→17
15989 measured reflections	*l* = −16→16

### Refinement

**Table:**

Refinement on *F*^2^	Primary atom site location: structure-invariant direct methods
Least-squares matrix: full	Secondary atom site location: difference Fourier map
*R*[*F*^2^ > 2σ(*F*^2^)] = 0.049	Hydrogen site location: inferred from neighbouring sites
*wR*(*F*^2^) = 0.136	H-atom parameters constrained
*S* = 1.08	*w* = 1/[σ^2^(*F*_o_^2^) + (0.0659*P*)^2^ + 0.2655*P*] where *P* = (*F*_o_^2^ + 2*F*_c_^2^)/3
3912 reflections	(Δ/σ)_max_ < 0.001
209 parameters	Δρ_max_ = 0.36 e Å^−^^3^
0 restraints	Δρ_min_ = −0.26 e Å^−^^3^

### Special details

**Table:**

Geometry. All e.s.d.'s (except the e.s.d. in the dihedral angle between two l.s. planes) are estimated using the full covariance matrix. The cell e.s.d.'s are taken into account individually in the estimation of e.s.d.'s in distances, angles and torsion angles; correlations between e.s.d.'s in cell parameters are only used when they are defined by crystal symmetry. An approximate (isotropic) treatment of cell e.s.d.'s is used for estimating e.s.d.'s involving l.s. planes.
Refinement. Refinement of *F*^2^ against ALL reflections. The weighted *R*-factor *wR* and goodness of fit *S* are based on *F*^2^, conventional *R*-factors *R* are based on *F*, with *F* set to zero for negative *F*^2^. The threshold expression of *F*^2^ > σ(*F*^2^) is used only for calculating *R*-factors(gt) *etc*. and is not relevant to the choice of reflections for refinement. *R*-factors based on *F*^2^ are statistically about twice as large as those based on *F*, and *R*- factors based on ALL data will be even larger.

### Fractional atomic coordinates and isotropic or equivalent isotropic displacement parameters (Å^2^)

**Table:**

	*x*	*y*	*z*	*U*_iso_*/*U*_eq_	
C10	0.88179 (15)	0.25846 (10)	0.48356 (11)	0.0372 (3)	
O3	0.69984 (11)	0.51832 (9)	0.73210 (10)	0.0539 (3)	
N2	0.99132 (14)	0.23098 (11)	0.47668 (11)	0.0514 (4)	
C11	0.33488 (15)	0.36674 (12)	0.54335 (13)	0.0472 (4)	
H11A	0.3265	0.4021	0.6069	0.071*	
H11B	0.2856	0.3046	0.5463	0.071*	
H11C	0.2989	0.4074	0.4885	0.071*	
N3	0.67042 (14)	0.18281 (10)	0.35965 (10)	0.0444 (3)	
H3A	0.6055	0.1592	0.3220	0.053*	
H3B	0.7520	0.1632	0.3503	0.053*	
O1	0.41901 (10)	0.06974 (7)	0.36195 (8)	0.0411 (3)	
N1	0.51316 (11)	0.27979 (8)	0.44545 (9)	0.0346 (3)	
O2	0.56463 (12)	−0.12848 (9)	0.36431 (9)	0.0530 (3)	
H2	0.6107	−0.0779	0.3565	0.079*	
C1	0.64429 (14)	0.25077 (10)	0.43342 (10)	0.0338 (3)	
C2	0.74686 (13)	0.29353 (10)	0.49497 (10)	0.0336 (3)	
C5	0.48117 (14)	0.34495 (10)	0.52525 (11)	0.0352 (3)	
C12	0.82613 (14)	0.41643 (10)	0.62401 (11)	0.0355 (3)	
H12	0.9135	0.3970	0.6085	0.043*	
C4	0.57932 (14)	0.38748 (10)	0.58437 (11)	0.0353 (3)	
H4	0.5543	0.4309	0.6365	0.042*	
C3	0.72004 (14)	0.36870 (10)	0.57037 (10)	0.0328 (3)	
C13	0.81221 (14)	0.49139 (10)	0.69951 (11)	0.0355 (3)	
C14	0.93855 (14)	0.54230 (11)	0.74837 (11)	0.0374 (3)	
C7	0.33697 (15)	0.15161 (11)	0.39359 (12)	0.0425 (3)	
H7A	0.3218	0.1471	0.4665	0.051*	
H7B	0.2500	0.1491	0.3580	0.051*	
C6	0.40756 (16)	0.24924 (11)	0.36958 (12)	0.0417 (3)	
H6A	0.4486	0.2430	0.3032	0.050*	
H6B	0.3404	0.3023	0.3645	0.050*	
C8	0.36595 (15)	−0.02510 (11)	0.39327 (12)	0.0417 (3)	
H8A	0.2689	−0.0262	0.3816	0.050*	
H8B	0.3840	−0.0349	0.4658	0.050*	
C9	0.43021 (16)	−0.10829 (11)	0.33368 (12)	0.0445 (4)	
H9A	0.3772	−0.1691	0.3414	0.053*	
H9B	0.4280	−0.0905	0.2617	0.053*	
C16	0.95355 (17)	0.50352 (15)	0.85821 (13)	0.0536 (4)	
H16A	1.0312	0.5340	0.8908	0.080*	
H16B	0.9646	0.4318	0.8574	0.080*	
H16C	0.8742	0.5205	0.8954	0.080*	
C17	0.91462 (18)	0.65630 (12)	0.75167 (14)	0.0517 (4)	
H17A	0.9923	0.6888	0.7821	0.078*	
H17B	0.8367	0.6703	0.7917	0.078*	
H17C	0.8999	0.6811	0.6833	0.078*	
C15	1.06820 (15)	0.52174 (14)	0.69025 (14)	0.0508 (4)	
H15A	1.1427	0.5551	0.7241	0.076*	
H15B	1.0582	0.5466	0.6215	0.076*	
H15C	1.0850	0.4506	0.6887	0.076*	

### Atomic displacement parameters (Å^2^)

**Table:**

	*U*^11^	*U*^22^	*U*^33^	*U*^12^	*U*^13^	*U*^23^
C10	0.0403 (8)	0.0336 (7)	0.0380 (7)	−0.0010 (6)	0.0054 (6)	−0.0014 (5)
O3	0.0340 (6)	0.0642 (7)	0.0635 (7)	0.0013 (5)	0.0022 (5)	−0.0277 (6)
N2	0.0414 (7)	0.0528 (8)	0.0601 (9)	0.0073 (6)	0.0055 (6)	−0.0048 (6)
C11	0.0335 (8)	0.0464 (8)	0.0617 (10)	0.0041 (7)	−0.0018 (7)	−0.0061 (7)
N3	0.0469 (7)	0.0444 (7)	0.0420 (7)	0.0025 (6)	0.0010 (5)	−0.0098 (5)
O1	0.0404 (6)	0.0358 (5)	0.0474 (6)	−0.0015 (4)	0.0056 (4)	0.0000 (4)
N1	0.0363 (6)	0.0318 (5)	0.0353 (6)	0.0008 (5)	−0.0042 (5)	0.0015 (4)
O2	0.0527 (7)	0.0528 (6)	0.0537 (7)	0.0076 (5)	0.0085 (5)	0.0170 (5)
C1	0.0390 (7)	0.0301 (6)	0.0322 (7)	0.0010 (6)	0.0029 (5)	0.0039 (5)
C2	0.0337 (7)	0.0312 (6)	0.0360 (7)	0.0010 (5)	0.0041 (5)	0.0018 (5)
C5	0.0341 (7)	0.0309 (6)	0.0404 (7)	0.0030 (5)	−0.0013 (5)	0.0018 (5)
C12	0.0294 (6)	0.0374 (7)	0.0395 (7)	0.0022 (6)	0.0008 (5)	−0.0001 (5)
C4	0.0343 (7)	0.0331 (6)	0.0385 (7)	0.0019 (6)	0.0019 (5)	−0.0025 (5)
C3	0.0349 (7)	0.0301 (6)	0.0335 (7)	0.0013 (5)	0.0016 (5)	0.0038 (5)
C13	0.0324 (7)	0.0383 (7)	0.0359 (7)	0.0016 (6)	−0.0004 (5)	0.0016 (5)
C14	0.0342 (7)	0.0428 (7)	0.0349 (7)	−0.0036 (6)	−0.0031 (5)	0.0029 (6)
C7	0.0359 (7)	0.0435 (8)	0.0477 (9)	0.0030 (6)	−0.0074 (6)	−0.0056 (6)
C6	0.0455 (8)	0.0386 (7)	0.0403 (8)	0.0045 (6)	−0.0127 (6)	0.0014 (6)
C8	0.0373 (8)	0.0412 (8)	0.0465 (9)	−0.0077 (6)	0.0027 (6)	0.0021 (6)
C9	0.0488 (9)	0.0391 (8)	0.0458 (8)	−0.0069 (7)	0.0027 (6)	0.0019 (6)
C16	0.0482 (9)	0.0698 (11)	0.0423 (9)	−0.0093 (8)	−0.0081 (7)	0.0114 (8)
C17	0.0525 (10)	0.0454 (9)	0.0570 (10)	−0.0091 (7)	−0.0052 (7)	−0.0010 (7)
C15	0.0340 (8)	0.0648 (10)	0.0536 (10)	−0.0091 (7)	0.0002 (7)	−0.0037 (8)

### Geometric parameters (Å, º)

**Table:**

C10—N2	1.1497 (19)	C4—H4	0.9300
C10—C2	1.4259 (19)	C13—C14	1.547 (2)
O3—C13	1.2526 (17)	C14—C16	1.529 (2)
C11—C5	1.5011 (19)	C14—C15	1.531 (2)
C11—H11A	0.9600	C14—C17	1.534 (2)
C11—H11B	0.9600	C7—C6	1.510 (2)
C11—H11C	0.9600	C7—H7A	0.9700
N3—C1	1.3504 (18)	C7—H7B	0.9700
N3—H3A	0.8600	C6—H6A	0.9700
N3—H3B	0.8600	C6—H6B	0.9700
O1—C7	1.4247 (18)	C8—C9	1.502 (2)
O1—C8	1.4289 (17)	C8—H8A	0.9700
N1—C1	1.3670 (17)	C8—H8B	0.9700
N1—C5	1.3974 (18)	C9—H9A	0.9700
N1—C6	1.4811 (18)	C9—H9B	0.9700
O2—C9	1.407 (2)	C16—H16A	0.9600
O2—H2	0.8200	C16—H16B	0.9600
C1—C2	1.4012 (19)	C16—H16C	0.9600
C2—C3	1.4323 (18)	C17—H17A	0.9600
C5—C4	1.351 (2)	C17—H17B	0.9600
C12—C3	1.401 (2)	C17—H17C	0.9600
C12—C13	1.411 (2)	C15—H15A	0.9600
C12—H12	0.9300	C15—H15B	0.9600
C4—C3	1.4312 (19)	C15—H15C	0.9600
			
N2—C10—C2	178.37 (16)	O1—C7—C6	109.03 (12)
C5—C11—H11A	109.5	O1—C7—H7A	109.9
C5—C11—H11B	109.5	C6—C7—H7A	109.9
H11A—C11—H11B	109.5	O1—C7—H7B	109.9
C5—C11—H11C	109.5	C6—C7—H7B	109.9
H11A—C11—H11C	109.5	H7A—C7—H7B	108.3
H11B—C11—H11C	109.5	N1—C6—C7	114.79 (12)
C1—N3—H3A	120.0	N1—C6—H6A	108.6
C1—N3—H3B	120.0	C7—C6—H6A	108.6
H3A—N3—H3B	120.0	N1—C6—H6B	108.6
C7—O1—C8	112.00 (11)	C7—C6—H6B	108.6
C1—N1—C5	119.52 (11)	H6A—C6—H6B	107.5
C1—N1—C6	120.22 (11)	O1—C8—C9	109.74 (12)
C5—N1—C6	120.06 (11)	O1—C8—H8A	109.7
C9—O2—H2	109.5	C9—C8—H8A	109.7
N3—C1—N1	117.96 (13)	O1—C8—H8B	109.7
N3—C1—C2	122.20 (13)	C9—C8—H8B	109.7
N1—C1—C2	119.82 (12)	H8A—C8—H8B	108.2
C1—C2—C10	118.41 (12)	O2—C9—C8	113.71 (13)
C1—C2—C3	122.30 (12)	O2—C9—H9A	108.8
C10—C2—C3	119.26 (12)	C8—C9—H9A	108.8
C4—C5—N1	120.90 (12)	O2—C9—H9B	108.8
C4—C5—C11	120.87 (13)	C8—C9—H9B	108.8
N1—C5—C11	118.24 (12)	H9A—C9—H9B	107.7
C3—C12—C13	125.83 (13)	C14—C16—H16A	109.5
C3—C12—H12	117.1	C14—C16—H16B	109.5
C13—C12—H12	117.1	H16A—C16—H16B	109.5
C5—C4—C3	122.91 (13)	C14—C16—H16C	109.5
C5—C4—H4	118.5	H16A—C16—H16C	109.5
C3—C4—H4	118.5	H16B—C16—H16C	109.5
C12—C3—C4	125.30 (12)	C14—C17—H17A	109.5
C12—C3—C2	120.75 (12)	C14—C17—H17B	109.5
C4—C3—C2	113.95 (12)	H17A—C17—H17B	109.5
O3—C13—C12	122.76 (13)	C14—C17—H17C	109.5
O3—C13—C14	116.80 (12)	H17A—C17—H17C	109.5
C12—C13—C14	120.42 (12)	H17B—C17—H17C	109.5
C16—C14—C15	109.94 (13)	C14—C15—H15A	109.5
C16—C14—C17	108.55 (14)	C14—C15—H15B	109.5
C15—C14—C17	108.78 (13)	H15A—C15—H15B	109.5
C16—C14—C13	107.47 (12)	C14—C15—H15C	109.5
C15—C14—C13	113.36 (12)	H15A—C15—H15C	109.5
C17—C14—C13	108.64 (12)	H15B—C15—H15C	109.5

### Hydrogen-bond geometry (Å, º)

Cg1 is the centroid of the dihydropyridine ring.

**Table:**

*D*—H···*A*	*D*—H	H···*A*	*D*···*A*	*D*—H···*A*
N3—H3*A*···O1	0.86	2.27	2.9083 (17)	131
C4—H4···O3	0.93	2.21	2.8405 (17)	124
O2—H2···O3^i^	0.82	2.03	2.650 (2)	132
C7—H7*A*···O2^ii^	0.97	2.47	3.303 (2)	144
C9—H9*A*···*Cg*1^ii^	0.97	2.81	3.5275 (16)	131

Symmetry codes: (i) *x*, −*y*+1/2, *z*−1/2; (ii) −*x*+1, −*y*, −*z*+1.

## References

[bb1] Farrugia, L. J. (1997). *J. Appl. Cryst.* **30**, 565.

[bb2] Ha, K., Heo, J. & Kim, H. J. (2009). *Acta Cryst.* E**65**, o3131. 10.1107/S1600536809048892 PMC2971777 21578854

[bb3] Kim, Y. H., Kim, H. J., Otgonbaatar, E. & Kwak, C.-H. (2011). *Acta Cryst.* E**67**, o670. 10.1107/S1600536811005587 PMC3052154 21522419

[bb4] Rigaku (2008). *RAPIDO-AUTO* Rigaku Corporation, Tokyo, Japan.

[bb5] Sheldrick, G. M. (2008). *Acta Cryst.* A**64**, 112–122. 10.1107/S0108767307043930 18156677

[bb6] VanAllan, J. A. & Reynolds, G. A. (1971). *J. Heterocycl. Chem.* **8**, 367–371.

